# Pd nanoparticles decorated on a porous Co(BDC-NH_2_) MOF as an effective heterogeneous catalyst for dye reduction

**DOI:** 10.1039/d3na00379e

**Published:** 2023-09-25

**Authors:** Hassan Keypour, Jamal Kouhdareh, Khadijeh Rabiei, İdris Karakaya, Rahman Karimi-Nami, Sedigheh Alavinia

**Affiliations:** a Department of Inorganic Chemistry, Faculty of Chemistry, Bu-Ali Sina University Hamedan 6517838683 Iran haskey1@yahoo.com; b Department of Chemistry, Faculty of Science, Qom University of Technology Qom Iran; c Department of Chemistry, College of Basic Sciences, Gebze Technical University 41400 Gebze Turkey; d Department of Chemistry, Faculty of Science, University of Maragheh Maragheh 55181-83111 Iran

## Abstract

Herein, a new catalytic nanocomposite [Co(BDC-NH_2_)-Pd NPs] composed of a Co(BDC-NH_2_) MOF has been developed. The catalyst was prepared by modifying the synthesized porous Co(BDC-NH_2_) MOF with decorated Pd nanoparticles. This nanocatalyst was used as a heterogeneous catalyst in the reductive degradation of organic dyes Rhodamine B and methyl orange with NaBH_4_. The kinetic and thermodynamic parameters of the reactions were evaluated. The results showed that the low catalyst content could successfully catalyze the dye reduction reaction quickly (1 min). The metal–organic frameworks unique porous morphology of the Co(BDC-NH_2_) MOF appears to increase dye adsorption and achieve effective dye reduction. Additionally, recyclability studies of the catalyst confirmed that it could be recovered and reused for 10 consecutive reaction cycles with negligible Pd leaching and reduction in catalytic activity.

## Abstract 2

Herein, a new catalytic nanocomposite [Co(BDC-NH_2_)-Pd NPs] composed of a Co(BDC-NH_2_) MOF has been developed.

## Graphical abstract

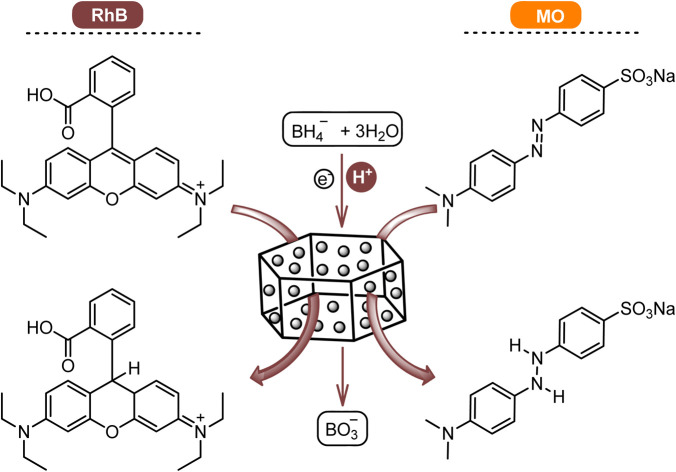

## 1 Introduction

Metal organic frameworks (MOFs) are produced by linking inorganic and organic units through strong bonds (lattice synthesis). Metal ions form the nodes that connect the arms of the linker in a repeating cage-like structure. Due to this hollow structure, MOFs have a unique large internal surface area.^1^ Nowadays, MOFs are considered to be good candidates for synthesizing supported catalysts, as science and technology today emphasize using sustainable processes and materials.^2–4^

Numerous studies have demonstrated the catalytic application and synthesis of different MOF derivatives with post-immobilized metal ions, pre-modified ligands, and metal nanoparticles (NPs). Several post-synthetic modification (PSM) methods have been described for functionalizing the organic carriers and secondary building units (SBUs) of MOFs.^5^

The production and use of dyes in industrial processes have increased dramatically. This causes the formation of wastewater from textile, paint, food, and other industries. Wastewater from these industries is frequently discharged into natural water bodies, causing severe water and environmental pollution and seriously threatening the health of humans and other species. The ongoing climate change is also affecting water availability for people around the world. Therefore, advanced treatment and removal of harmful pollutants from municipal and industrial wastewater is becoming increasingly important. Removing dyes from wastewater can be achieved in various ways, including physical, chemical, and biological treatments.^6^

Toxic dyes impair photosynthesis and inhibit plant growth by increasing biochemical and chemical oxygen demand. Moreover, they enter the food chain, causing recalcitrance and bioaccumulation and lowering the aesthetic quality of water bodies, potentially promoting toxicity, mutagenicity, and carcinogenicity. Given the importance of environmental issues and regulations, developing various methods such as discoloration and degradation, photo, electron degradation, and adsorption on potential adsorbents has received significant attention. Therefore, it is imperative to effectively treat dyes containing wastewater using environmentally friendly technologies to avoid negative impacts on the environment, human health, and natural water resources. There is an urgent need to find the most appropriate strategies to successfully degrade or remove dyes from wastewater.^7–11^

Methyl orange (MO) and Rhodamine B (RhB) are hazardous dyes in industrial wastewater. One of the most promising approaches is the reductive bleaching of dyes, usually catalyzed by metal catalysts using reducing agents.^12^ The metal catalyst is generally stabilized on a support to make the process more efficient. Compared with homogeneous catalysts and unsupported metal nanoparticles, metal nanoparticle structures supported on high surface area supports are preferred in catalytic applications due to their easy separation, recovery, and relatively better reactivity.^13,14^ Various metal nanoparticles, such as Ni,^15^ Cu,^16^ and Ti,^17^ have been used in different organic reactions. In particular, Pd nanoparticles play a significant catalytic role by allowing easy contact with the reactants, improving their catalytic power.^18^ Notably, Pd nanoparticles can be used to improve and/or discover alternative methods for catalytic reduction/degradation of organic and inorganic pollutants in water/wastewater.^19,20^ This simplifies the recovery of the catalyst and increases its recyclability.^21^ On the other hand, depending on the support's characteristics, the catalytic process's efficiency can be increased. These disclosures demonstrate the urgency of designing and synthesizing catalysts with properties that will destroy dyes and toxic chemicals in industrial wastewater. Efficiency, stability, and economy are very important points in the design of these catalysts.

This study initially synthesized a Co(BDC-NH_2_) MOF with good catalytic substrate potential. Then, the catalyst [Co(BDC-NH_2_)-Pd NPs] was modified with decorated Pd nanoparticles and characterized using FT-IR, XRD, SEM, TGA, ICP-OES, EDXS, and BET analytical techniques. Finally, this catalyst was used for the reductive degradation of MO and RhB in aqueous media using NaBH_4_. To further evaluate the catalytic performance of the nanocomposite [Co(BDC-NH_2_)-Pd NPs], the kinetic and thermodynamic parameters of the reaction, including activation energy, enthalpy, and entropy of each degradation dye, and leaching and recyclability of Co(BDC-NH_2_)-Pd NPs were estimated.

## 2 Experimental

### 2.1. Catalyst preparation

**Scheme 1:**
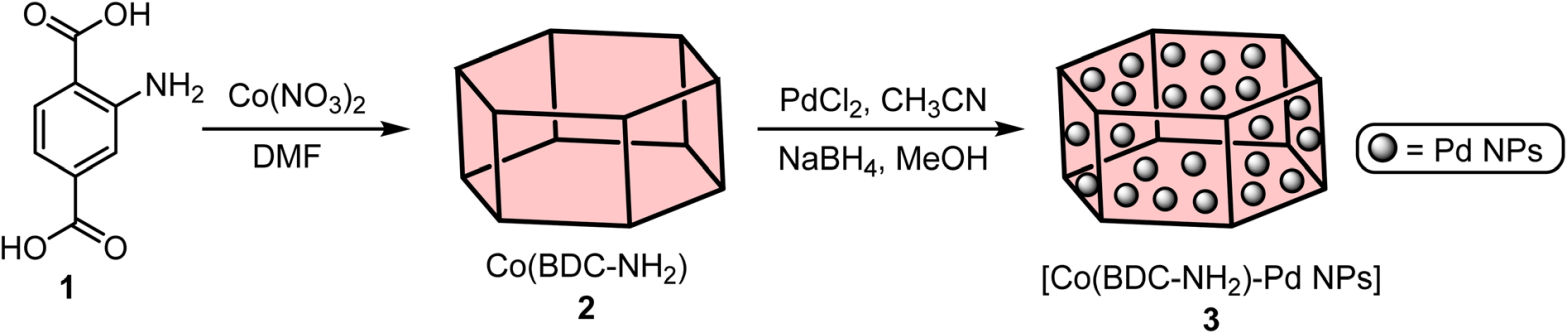
Preparation of the [Co(BDC-NH_2_)-Pd NPs] 3.

Co(BDC-NH_2_) 2 was prepared using a published method.^22–25^ 2-Aminoterephthalic acid (BDC-NH_2_) 1 (1.087 g) and cobalt(ii) nitrate (0.953 g) were added to a solution of dimethylformamide (DMF) (20 mL), transferred to a Teflon lined stainless steel autoclave and stirred for 30 min, followed by heating at 110 °C for 48 h. Next, Co(BDC-NH_2_) 2 was obtained after washing with EtOH and dried overnight at 60 °C. To impregnate Pd nanoparticles on the Co(BDC-NH_2_) support, PdCl_2_ (50 mg) was dissolved in acetonitrile (10 mL) and gently added to the agitating suspension of Co(BDC-NH_2_) 2 (1 g) in acetonitrile (30 mL). The mixture was stirred for 1 h at room temperature. After that, the reduction of Pd was carried out with NaBH_4_ in MeOH (10 mL, 0.2 M) under an Ar atmosphere for 1 h. Finally, [Co(BDC-NH_2_)-Pd NPs] 3 was collected, rinsed with methanol, and dried at room temperature. The synthesis procedure of [Co(BDC-NH_2_)-Pd NPs] 3 is displayed in Scheme 1.

### 2.2. Catalyst characterization

**Fig. 1:**
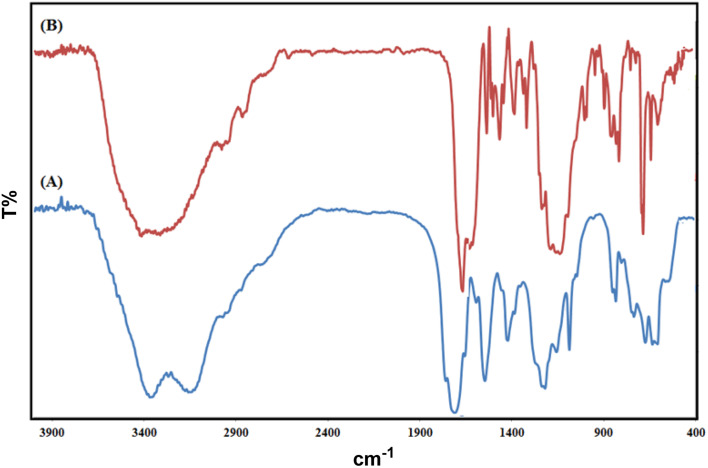
FT-IR spectroscopy results for Co(BDC-NH_2_) 2 (A) and [Co(BDC-NH_2_)-Pd NPs] catalyst 3 (B).

The Fourier transform infrared (FT-IR) spectroscopy results of Co(BDC-NH_2_) 2 and [Co(BDC-NH_2_)-Pd NPs] 3 are presented in Fig. 1. The FT-IR spectra of bare Co(BDC-NH_2_) 2 (Fig. 1A) show broad peaks at 1500–1600 and 3300–3500 cm^−1^, regarding the free and uncoordinated NH_2_ groups. Stretching vibrations of the C–N bond of H_2_BDC-NH_2_ show themselves at 1250 and 1350 cm^−1^. Shifts in all these peaks, shown in blue, indicate that the attachment of Pd to the structure of the catalyst [Co(BDC-NH_2_)-Pd NPs] 3 complexes is formed successfully (Fig. 1B). Other characteristic peaks of various parts of the composite overlapped. Other characterization methods were used to prove the formation of the catalyst.

**Fig. 2:**
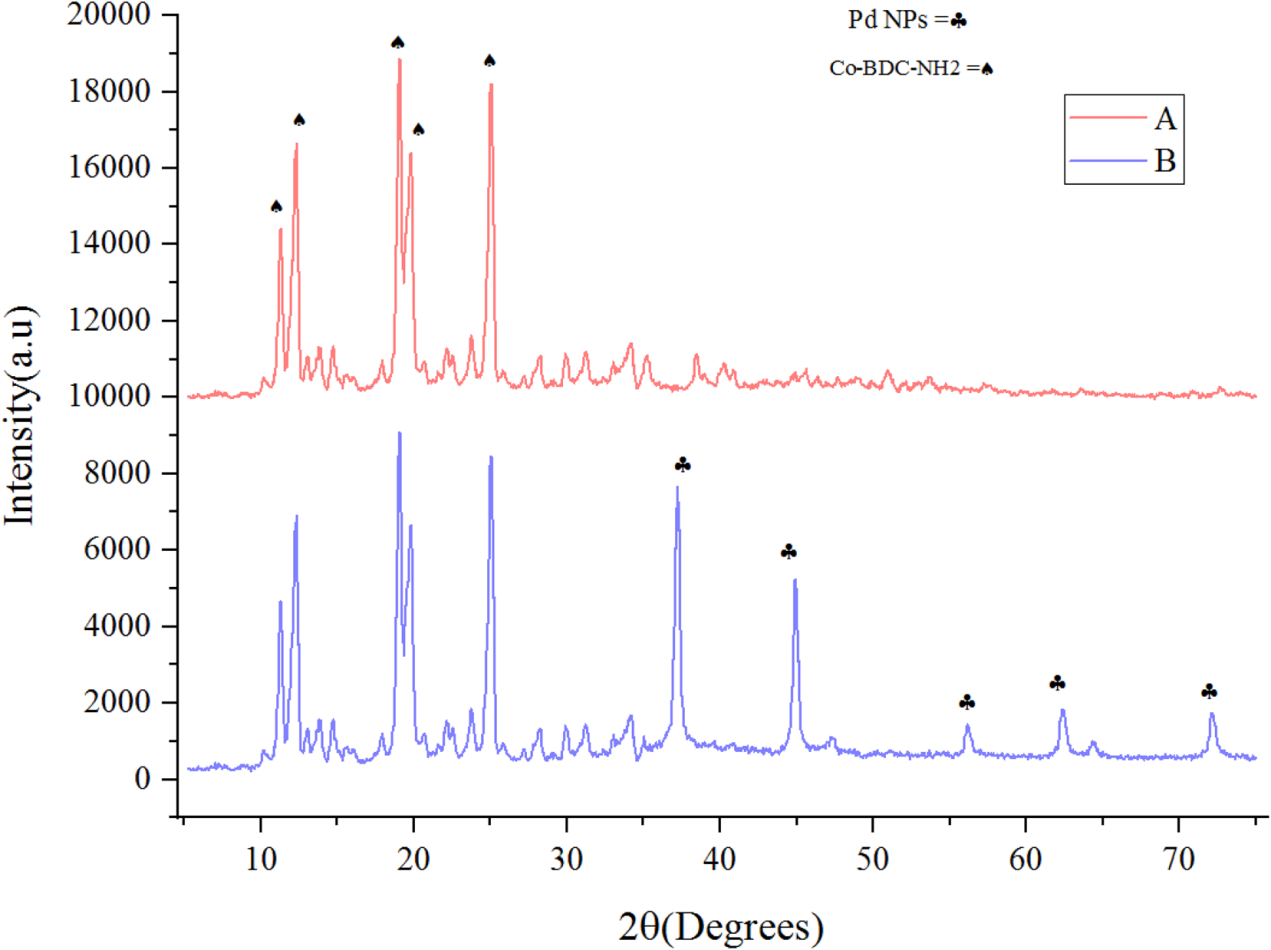
X-ray diffraction (XRD) patterns for Co(BDC-NH_2_) 2 (A) and [Co(BDC-NH_2_)-Pd NPs] catalyst 3 (B).

The crystalline structure of synthesized Co(BDC-NH_2_) 2 and [Co(BDC-NH_2_)-Pd NPs] 3 was investigated *via* an X-ray diffraction (XRD) technique (Fig. 2). The XRD pattern of bare Co(BDC-NH_2_) 2 reveals all characteristics, which prove its crystallinity and successful synthesis (Fig. 2A). The XRD patterns of Co(BDC-NH_2_) and [Co(BDC-NH_2_)-Pd NPs] 3, respectively, at 2*θ* = 11, 12, 17, 18, and 25°, indicate the preservation of the internal retention based on post synthesis changes of [Co(BDC-NH_2_)-Pd NPs] 3, corresponding to standard Bragg reflections (110), (210), (230), and (315) of the face-centered cubic lattice of Pd NPs (Fig. 2B). These spectra also exhibit all the characteristics of Co(BDC-NH_2_) 2, with a minor shift to higher 2*θ* which is a natural result of the composition,^26^ proving that the MOF preserves its crystalline structure throughout the whole synthesis process.

**Fig. 3:**
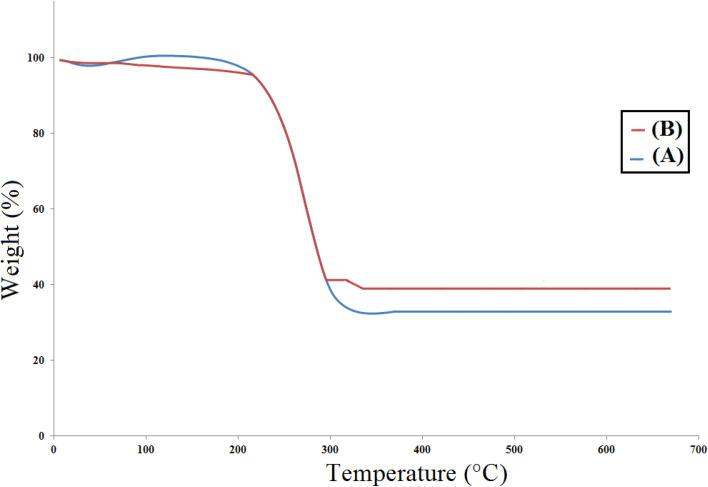
Thermogravimetric analysis (TGA) for Co(BDC-NH_2_) 2 (A) and [Co(BDC-NH_2_)-Pd NPs] catalyst 3 (B).

To check the thermal stability of catalyst [Co(BDC-NH_2_)-Pd NPs] 3, a thermogravimetric analysis (TGA) was done where the low weight loss of about 10% at low temperatures is related to the evaporation of solvents adsorbed on the catalyst structure. The organic substance, *i.e.* BDC-NH_2_, which was fixed on the Co(BDC-NH_2_) MOF, was decomposed at 250–500 °C, to an extent of 50% for Co(BDC-NH_2_) MOF 2 and 55% for [Co(BDC-NH_2_)-Pd NPs] 3. The last weight dissipation, which is less than 5%, may be related to the transformation of the thermal crystal phase of Pd nanoparticles on [Co(BDC-NH_2_)-Pd NPs] 3 (Fig. 3).^27^

**Fig. 4:**
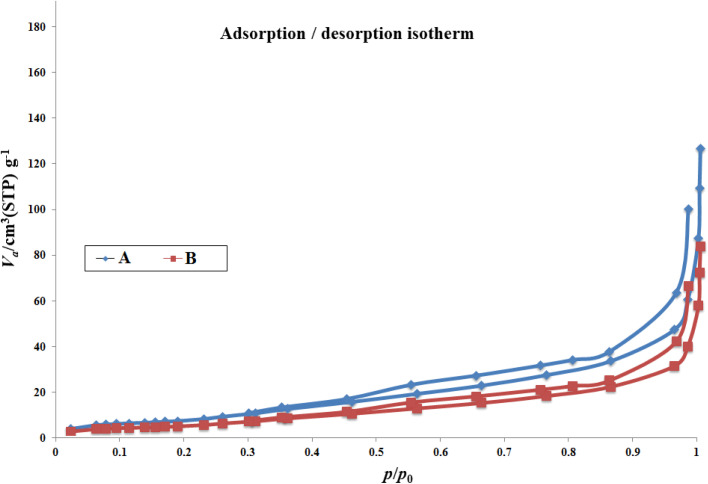
N_2_ adsorption–desorption isotherms for Co(BDC-NH_2_) 2 (A) and [Co(BDC-NH_2_)-Pd NPs] catalyst 3 (B).

N_2_ adsorption/desorption techniques were used to determine the surface structural parameters, and the results are plotted in Fig. 4. The surface area obtained based on the BET isotherm is 125.22 m^2^ g^−1^, and the total pore volume of the catalyst is 0.192 cm^3^ g^−1^. The adsorption isotherm is of type III, and the appearance of a hysteresis loop indicates the presence of mesopores in the sample.

**Fig. 5:**
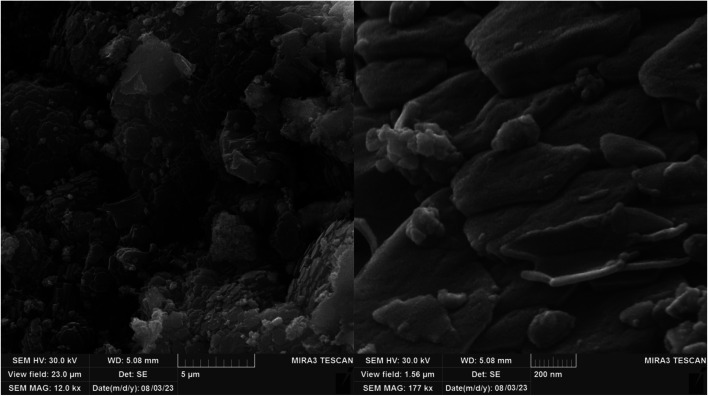
Scanning electron microscopy (SEM) images for [Co(BDC-NH_2_)-Pd NPs] catalyst 3.

Scanning electron microscopy (SEM) is a technique that uses a focused beam of high-energy electrons to identify nanomaterials that produce distinct signals on the surface of solid samples (Fig. 5). Receiving these signals and processing the information obtained from them causes the electrons to interact with the sample, revealing information about the sample such as external morphology (texture), material orientation, crystal structure, and chemical composition.^28^ In these images, crystal structures and palladium nanoparticles with modified ligands can be seen on the surfaces of these metal organic frameworks.

**Fig. 6:**
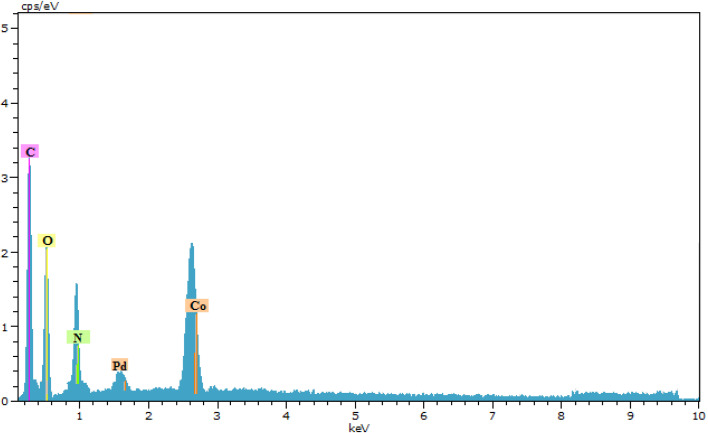
Energy-dispersive X-ray spectroscopy (EDXS) results for [Co(BDC-NH_2_)-Pd NPs] catalyst 3.

An energy-dispersive X-ray spectroscopy (EDXS) system is an accessory to electron microscope equipment (scanning electron microscope (SEM) or transmission electron microscope (TEM) equipment) and microscopic imaging capabilities. The spectrum generated by EDXS was analyzed with respect to the element peaks that make up the sample composition, giving the types of atoms present and the percentage of those atoms in the sample structure. The spectra of the synthesized nanocatalysts showed the presence of the elements C, N, O, Co, and Pd, which could signify the success of the desired synthesized complex (Fig. 6).^29^

### 2.3. Reductive degradation of dyes

The catalytic degradation of dyes was carried out according to a classical procedure.^30–32^ MO and RhB were used as organic dyes, and [Co(BDC-NH_2_)-Pd NPs] 3 and NaBH_4_ were employed as the catalyst and reducing agent, respectively. NaBH_4_ (0.3 mL, 0.1 M) and optimal levels of catalyst and dye (3 mL) were mixed and stirred at different temperatures. According to the results of the spectra UV visible light, [Co(BDC-NH_2_)-Pd NPs] 3 was collected, washed several times with EtOH : H_2_O (1 : 1), and dried.

## 3 Results and discussion

### 3.1. Catalytic activity

To study the activity of [Co(BDC-NH_2_) Pd NPs] 3 catalyst, it was decided to use NaBH_4_ for the reductive degradation of RhB and MO. Initially, the optimal loading of catalyst [Co(BDC-NH_2_)-Pd NPs] 3 for the reduction of each dye was evaluated (Table 1). Experimental data confirmed that the optimal catalyst loading [Co(BDC-NH_2_)-Pd NPs] 3 for each dye differed. A lower catalyst content [Co(BDC-NH_2_)-Pd NPs] 3 (10 mg) was required for MO reduction compared to RhB (15 mg).

**Table 1:** The optimization of the amount of [Co(BDC-NH_2_)-Pd NPs] 3 for reduction of MO and RhB

Dye	Catalyst amount (mg)	Conversion (%)
MO	5	80
	10	100
	15	100
RhB	5	63
	10	79
	15	100
	20	100

**Fig. 7:**
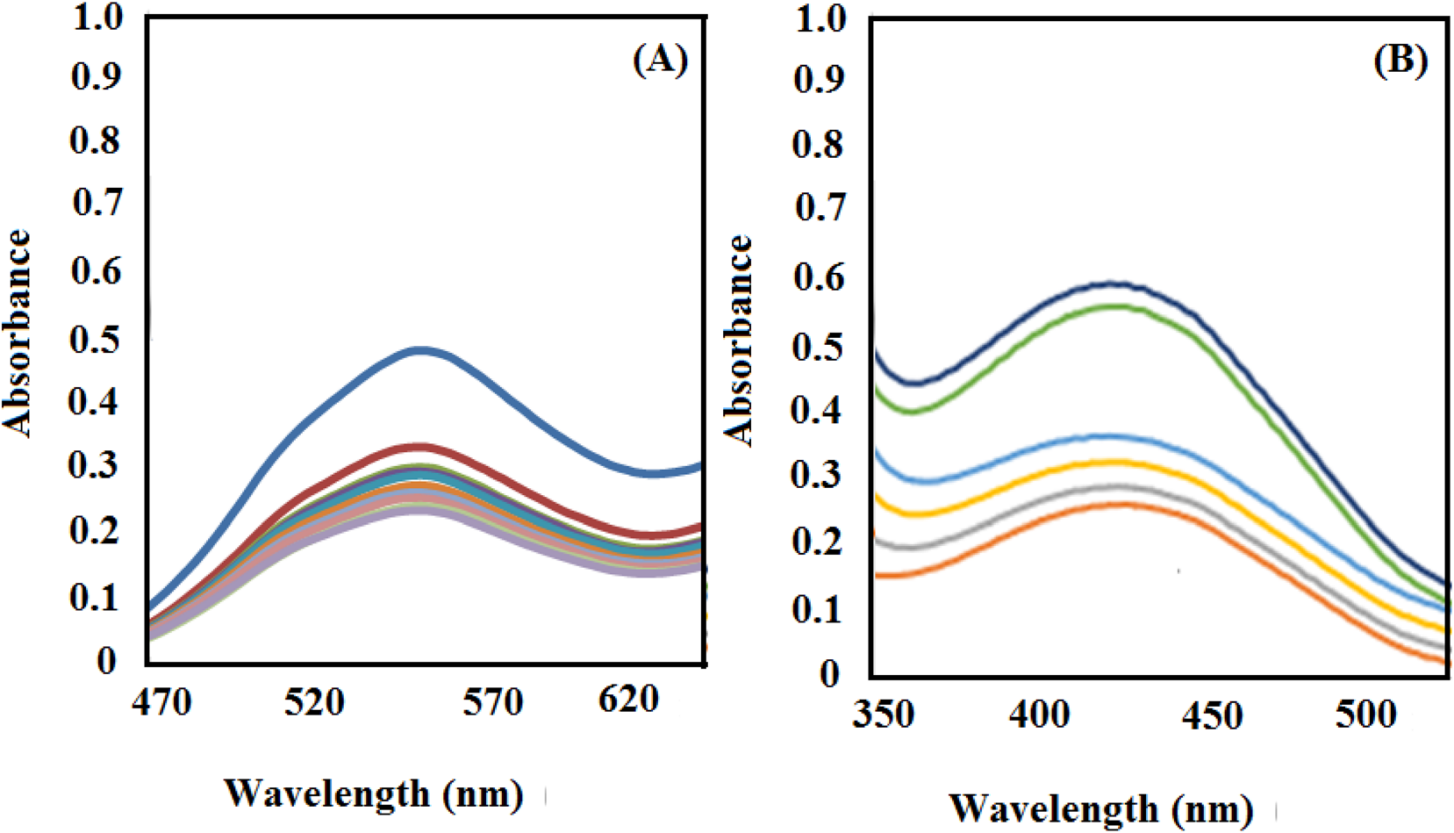
UV-vis spectra for reduction of MO (A) and RhB (B) dyes under [Co(BDC-NH_2_)-Pd NPs] 3.

Then, after the reduction process, the UV-vis spectrum of the dye was obtained as a function of time (Fig. 7). As proved by the decrement and disappearance of the distinctive bands of RhB (*λ*_max_ = 550 nm) and MO (*λ*_max_ = 465 nm), both dyes are degraded in aqueous media in the presence of NaBH_4_ and low content of [Co(BDC-NH_2_)-Pd NPs] in a very short reaction time (about 1 min).

The reduction rate constant (*k*_app_) was calculated for each dye in the next step. According to the literature, the mechanism of this process is assumed to be the Eley–Rideal mechanism.^33,34^ Considering pseudo-first-order kinetics,^35^ the reduction of each dye was performed at four different reaction temperatures (298, 303, 308, and 313 K). The equation of *k*_app_ can be evaluated using the equation below (eqn (1)).

1 ln *C*_*t*_/*C*_0_ = ln *A*_*t*_/*A*_0_ = −*k*_app_*t*

In this equation, *C*_*t*_ is the dye concentration at time = *t*, and *C*_0_ is the initial concentration. The concentration can be derived from the amount of dye absorbance at its *λ*_max_; that is, ln *C*_*t*_/*C*_0_ equals ln *A*_*t*_/*A*_0_. Hence, the *k*_app_ value is simply obtained from the slope of the ln(*A*_*t*_/*A*_0_) *versus t* (s) plot (Table 2, table caption: “Kinetic and thermodynamic parameters of the reduction of RhB and MO in the presence of [Co(BDC-NH_2_)-Pd NPs] 3”). As listed, upon increasing the reaction temperature, the *k*_app_ value increased. This is justified by the more effective collision of the reagents at higher temperatures.

Then, to estimate the activation energy (*E*_a_) for the reductive degradation of MO and RhB, the Arrhenius equation (eqn (2)) was applied.

2 ln *k* = ln *A* − (*E*_a_/*RT*)

**Fig. 8:**
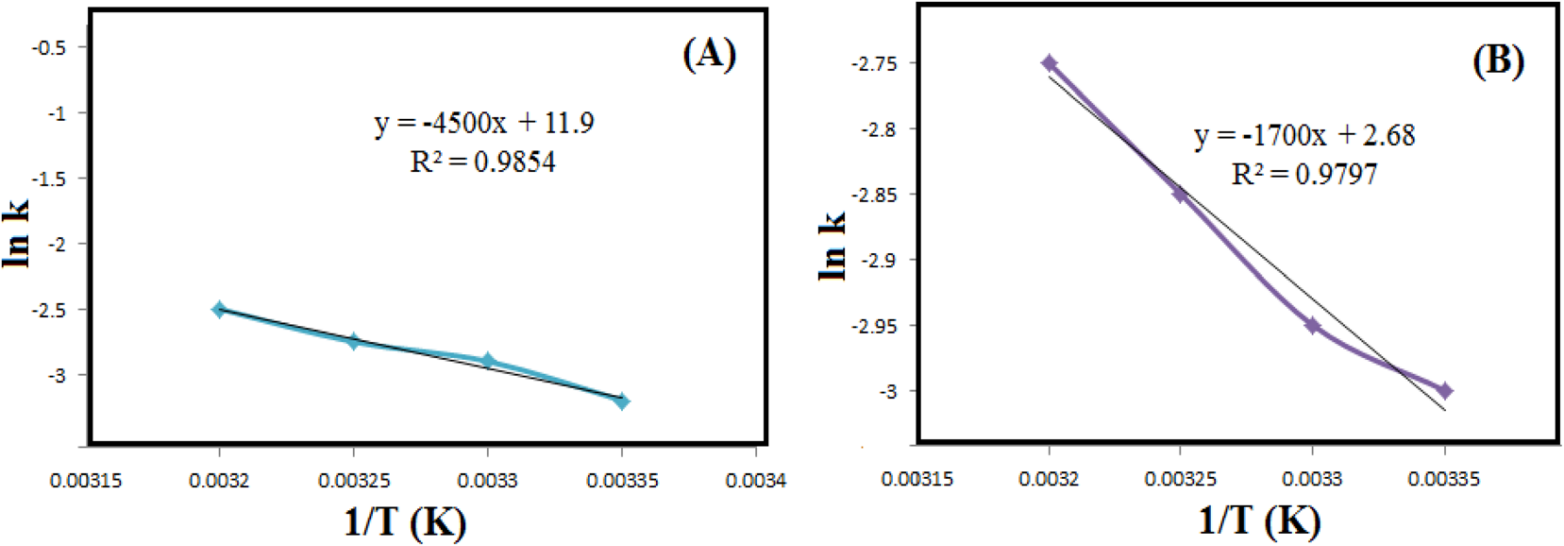
The diagrams of ln *k versus* 1/*T* for reductive degradation of RhB (A) and MO (B) at different temperatures.

With a value of *k*, *E*_a_ is obtained from the slope of ln *k* as a function of 1/*T* (Fig. 8 and Table 2). Applying the Eyring equation (eqn (3)), the thermodynamic parameters (Δ*S*^#^ and Δ*H*^#^) of the reduction degradation reaction for both dyes are assessed.

3 ln(*k*/*T*) = ln(*k*_B_/*h*) + Δ*S*^#^/*R* − Δ*H*^#^/*R*(1/*T*)

**Fig. 9:**
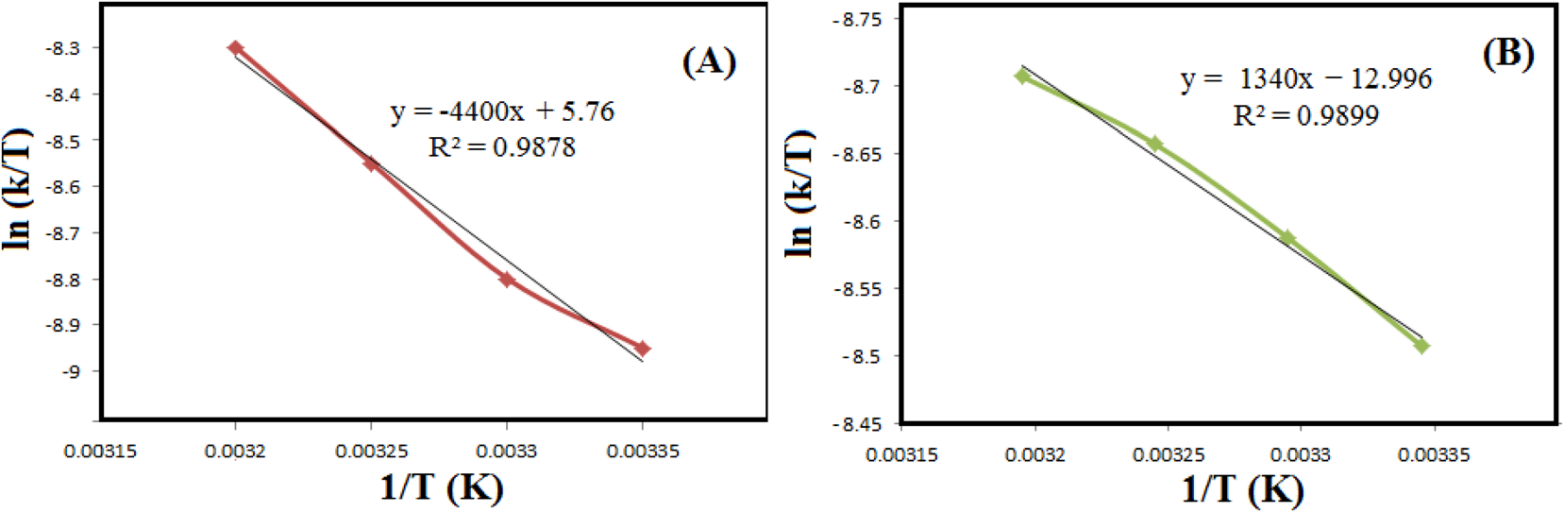
The diagrams of ln(*k*/*T*) *versus* 1/*T* for reductive degradation of RhB (A) and MO (B) at different temperatures.

Since Boltzmann's constant (*k*_B_) and Planck's constant (*h*) are constant values, Δ*H*^#^ and Δ*S*^#^ can be measured from the slope. The sequester of the plot of ln(*k*/*T*) *versus* 1/*T*, respectively (Fig. 9). As summarized in Table 2, the Δ*H*^#^ values for RhB and MO reduction were 10.7 and 34.9 J mol K^−1^, respectively. The Δ*S*^#^ values for RhB and MO reduction were estimated to be −89.9 and −154.3 J mol K^−1^, respectively.

**Fig. 10:**
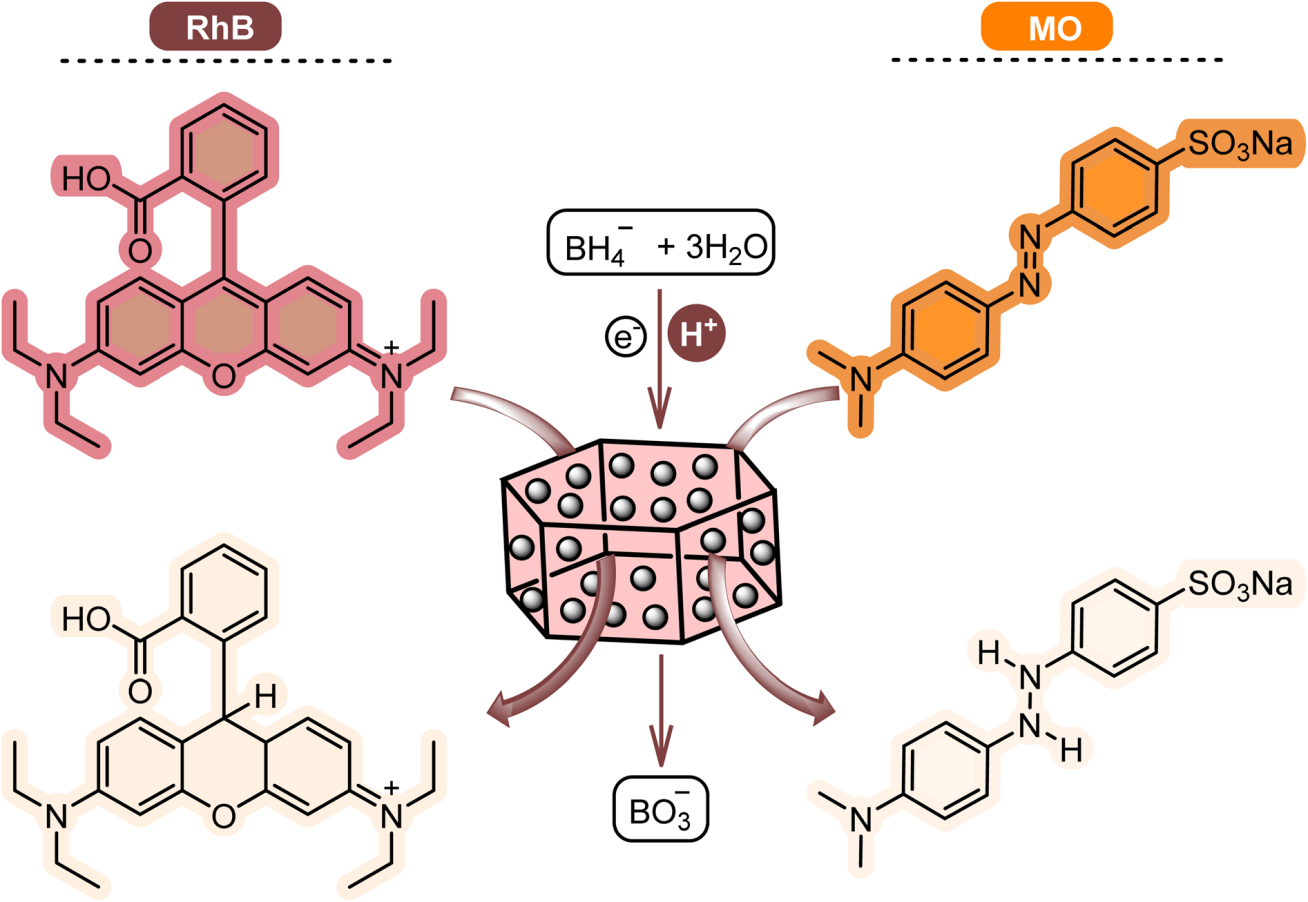
The reductive degradation of RhB and MO in the presence of [Co(BDC-NH_2_)-Pd NPs] 3.

Considering the previous reports, the proposed mechanism of [Co(BDC-NH_2_)-Pd NPs] 3 assisted dye degradation can be explained as follows.^21^ Initially, NaBH_4_ dissociates to generate borohydride ions, which are adsorbed on [Co(BDC-NH_2_)-Pd NPs] 3 surfaces (Fig. 10). In addition to borohydride ions, RhB or MO are also adsorbed *via* non-covalent interactions such as π–π stacking. In the next step, the generated hydride ions are transferred to the dyes on the surface of the catalyst and facilitate reduction. Finally, the [Co(BDC-NH_2_)-Pd NPs] 3 catalyst is desirable for degrading the mentioned pigments.

The efficiency of [Co(BDC-NH_2_)-Pd NPs] 3 was determined by comparison with other catalytic systems (Table 3). The time to complete the reaction is much less than with other catalysts, which is one of the advantages of the mentioned catalyst.

**Table 2:** Kinetic and thermodynamic parameters of the reduction of RhB and MO in the presence of [Co(BDC-NH_2_)-Pd NPs] 3

Dye	*T* (K)	*k* (min^−1^)	*E*_a_ (kJ mol^−1^)	Δ*H*^#^ (kJ mol^−1^)	Δ*S*^#^ (J mol K^−1^)
RhB	298	0.05	11.9	10.7	−89.9
	303	0.05			
	308	0.05			
	313	0.06			
MO	298	0.03	38.2	34.9	−154.3
	303	0.05			
	308	0.06			
	313	0.07			

**Table 3:** Comparison of various catalysts in the reduction of RhB and MO

Substrate	Catalyst	Time	Ref.
RhB	SiNWAs–Cu	14 min	36
RhB	Fe_3_O_4_@PANI@Au	18 min	37
RhB	Au-PANI nanocomposite	15 min	38
RhB	Fe_3_O_4_/Ag	15 min	39
RhB	Ag/HLaNb_2_O_7_	47 min	40
RhB	PS/Ag	10 min	41
RhB	Copper nanocrystals	5 min	42
RhB	Natrolite zeolite/Pd nanocomposite	8 s	43
RhB	[Co(BDC-NH_2_)-Pd NPs] 3	5 s	This work
MO	Cu@SBA-15	5 min	44
MO	Natrolite zeolite/Pd nanocomposite	2 min	43
MO	[Co(BDC-NH_2_)-Pd NPs] 3	5 s	This work

**Fig. 11:**
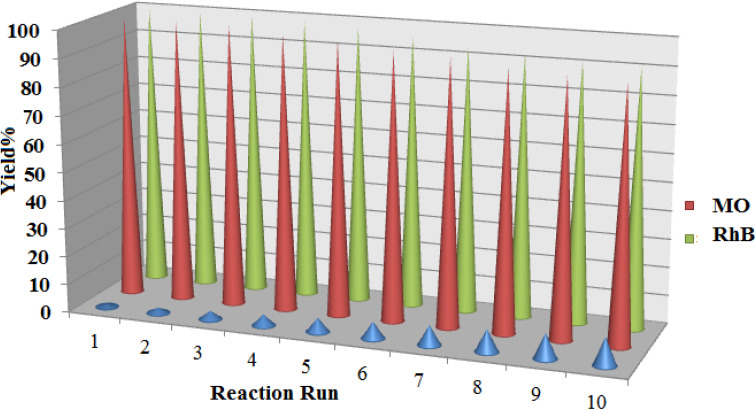
The recycling results for reduction of MO and RhB in the presence of [Co(BDC-NH_2_)-Pd NPs] 3.

In addition to the catalytic activity of [Co(BDC-NH_2_)-Pd NPs] 3, the recyclability, an essential characteristic of heterogeneous catalysts, was also evaluated. Given the importance of this project, the recyclability of the reaction of the two dyes has been questioned. Reassuringly, catalyst recovery is fast and easy due to the heterogeneous structure of [Co(BDC-NH_2_)-Pd NPs] 3. After washing and drying under conventional conditions, the recovered [Co(BDC-NH_2_)-Pd NPs] 3 was used for the next cycle. The recovered [Co(BDC-NH_2_)-Pd NPs] 3 was effective for both reactions and showed no reduction in activity in any of the three reactions (Fig. 11). In these recoveries, a slight loss of [Co(BDC-NH_2_)-Pd NPs] 3 activity was detected. With this decreasing trend, a decrease in the activity of the ten times recycled [Co(BDC-NH_2_)-Pd NPs] 3 catalyst from 100% to 90% was observed in the reduction of MO. After 10 trials, this value was 9% for the reduction of RhB.

**Fig. 12:**
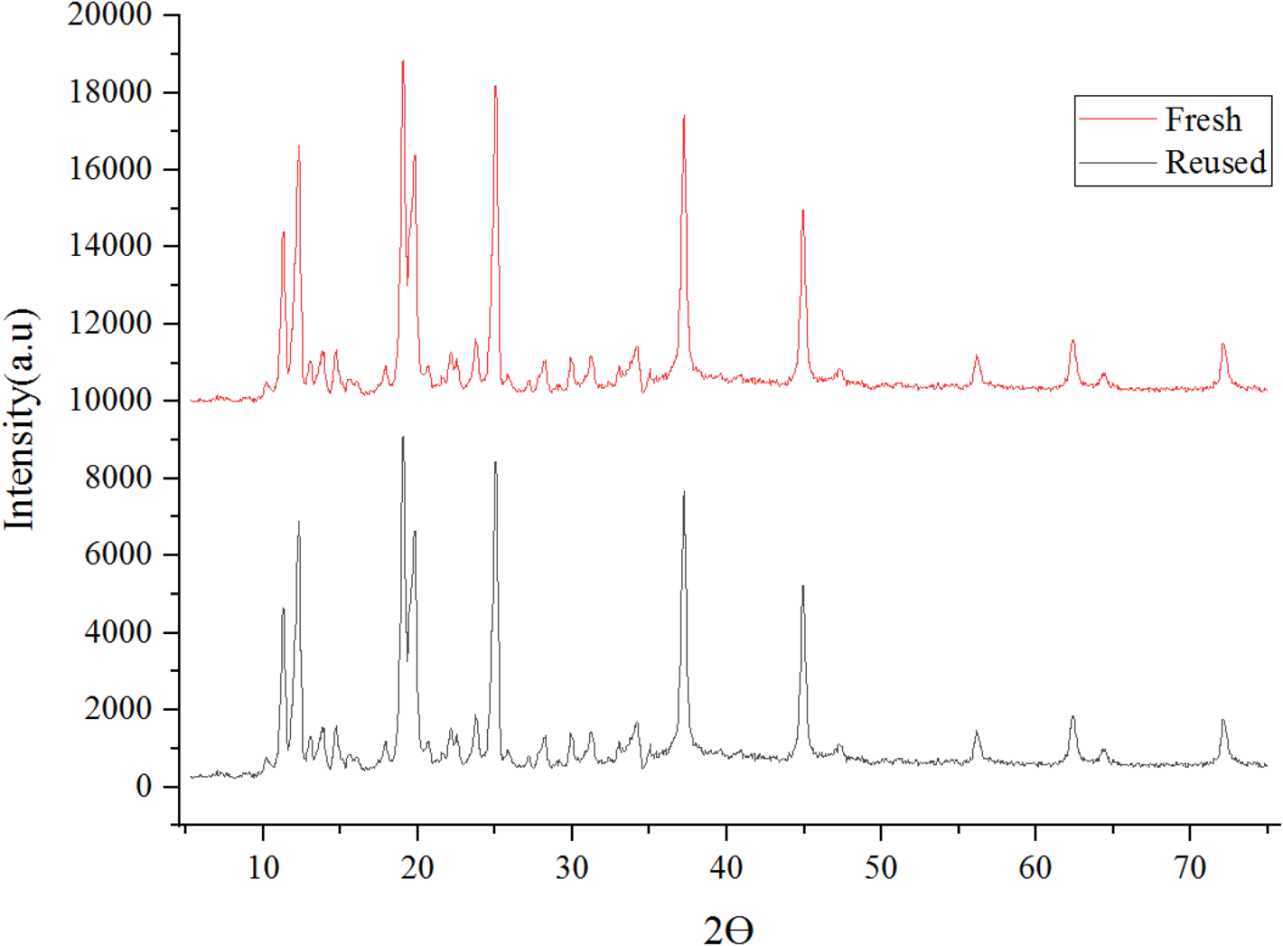
X-ray diffraction (XRD) patterns of fresh and reused [Co(BDC-NH_2_)-Pd NPs] catalyst 3.

**Fig. 13:**
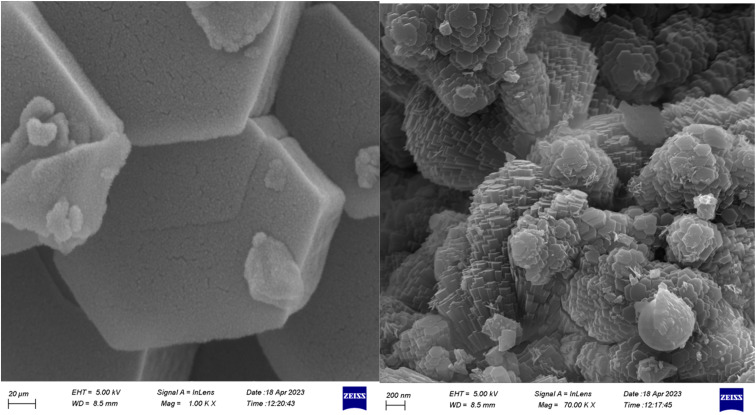
Scanning electron microscopy (SEM) images for reused [Co(BDC-NH_2_)-Pd NPs] catalyst 3.

The recovery of catalyst [Co(BDC-NH_2_)-Pd NPs] 3 in this study indicates a very efficient sequential application. The X-ray diffraction (XRD) (Fig. 12) and SEM (Fig. 13) image of the [Co(BDC-NH_2_)-Pd NPs] 3 catalyst were studied. The catalyst recovered after synthesis showed stability, and succeeded in preserving its structure. Thus, we can conclude that despite the impurity absorbed into the used catalyst, the original crystalline structure does not change during its use in the reaction. The Pd load in the material was found to be 7.52% and, after recovery, 7.39%, being estimated *via* the ICP-OES method.

Based on the theoretical and experimental investigations of these modified nanopolymers and the demonstration of their stability in the reactions, it is clear that this class of compounds can be used more widely.

## 4 Conclusion

In conclusion, for the preparation of the catalyst, Co(BDC-NH_2_) MOF 2 was first synthesized, and then the porous Co(BDC-NH_2_) MOF 2 was modified with decorated Pd nanoparticles. The catalytic performance of [Co(BDC-NH_2_)-Pd NPs] 3 on NaBH_4_ assisted reductive degradation of RhB and MO confirmed that [Co(BDC-NH_2_)-Pd NPs] 3 with low Pd loading could efficiently degrade the two dyes quickly (in about 1 min). After synthetic and thermodynamic studies, the synthesized catalyst was found to have excellent efficiency and ability to destroy dyes and toxins in wastewater. Since it is a heterogeneous catalyst, it can be easily separated and has a very durable structure. Notably, it has been demonstrated that [Co(BDC-NH_2_)-Pd NPs] 3 can be recovered and reused for 10 consecutive reaction cycles with negligible Pd leaching.

## Conflicts of interest

The authors declare that they have no competing interests.

## Author contributions

Prof. Hassan Keypour: conceptualization, methodology, resources, visualization, supervision, project administration. Dr Jamal Kouhdareh: validation, investigation, data curation, writing – original draft. Dr İdris Karakaya: validation, investigation, data curation, review & editing. Dr Rahman Karimi-Nami: validation, investigation, data curation, review & editing.

## Supplementary Material
